# Inflammatory Syndrome Regulated by WNT/β-Catenin, Non-Muscle Myosin and α-SMA Is a Self-Organized Dissipative Structure

**DOI:** 10.3390/biology15201882

**Published:** 2026-10-19

**Authors:** Yves Lecarpentier, Aurélien Morini, Christophe Locher, Xénophon Krokidis

**Affiliations:** 1 Centre de Recherche Clinique, Grand Hôpital de l’Est Francilien, 77100 Meaux, France; 2 Service d’Anatomie et Cytologie Pathologiques, Grand Hôpital de l’Est Francilien, 77600 Jossigny, France; amorini@ghef.fr; 3 Service d’Hépato-Gastro-Entérologie, Grand Hôpital de l’Est Francilien, 77100 Meaux, France; clocher@ghef.fr; 4 Xénomics, 75008 Paris, France; xenophon.krokidis@scienomics.com

**Keywords:** syndrome, inflammation, entropy production rate, excess entropy production, bifurcation, self-organization, dissipative structure

## Abstract

In medicine, several pathological processes have been characterized as dissipative structures. Until now, no medical syndrome had been classified as such. One of the most common—pulmonary inflammatory syndrome, characterized by redness, heat, swelling and pain—exhibits various properties required for the definition of a dissipative structure. It occurs in an open far-from-equilibrium system: its thermodynamic force varies non-linearly with its thermodynamic flow. Its excess entropy production is negative, conferring upon it the characteristics of a self-organized structure. Finally, it exhibits a bifurcation on the graph of entropy production rate plotted against a specific parameter (G, the curvature of the tension–velocity relationship). These characteristics are observed in various pulmonary diseases, such as cancer, tuberculosis, sarcoidosis, infection, etc. They share a common feature: the inflammatory syndrome that characterizes them all. The notable clinical signs are the activation of WNT/β-catenin, non-muscle myosin and α-SMA in newly formed myofibroblasts, which define the inflammatory syndrome.

## Simple Summary

Dissipative structures are common in physics, chemistry and even biology. Yet they are far less common in medicine. In the present study, the dissipative structures represent not a specific disease but a syndrome encountered in a vast number of diseases: pulmonary inflammatory syndrome, characterized by the activation of WNT/beta-catenin, non-muscle myosin, and alpha-SMA pathways.

## 1. Introduction

### 1.1. Inflammatory Syndrome

Inflammatory syndrome comprises both clinical and biological components. Locally, it is characterized by the seminal semiological tetrad of Aulus Cornelius Celsus (25 BC–AD 50): pain, swelling, redness and heat. He formulated a description of inflammation that remains valid today: “Notae vero inflammationis sunt quatuor: rubor et tumor cum calore et dolore” [1]. All living biological tissues undergoing an inflammatory process exhibit the hyperactivated WNT/β-catenin pathway. This activates the non-muscle myosin and renders the tissues contractile. When a disease possesses contractile properties, its energy E can be determined. Furthermore, living tissues are open systems that exchange energy and matter with their surroundings. They operate far -from- equilibrium when the thermodynamic force varies non-linearly with the thermodynamic flow. The thermodynamic force is E/T and its dimension is energy/T. The thermodynamic flow (vo) is the shortening velocity of the non-muscle myosin molecular motor. The product of thermodynamic force and thermodynamic flow is the entropy production rate. In far-from-equilibrium thermodynamics, the derivative of the entropy production rate is the excess entropy production. It can be positive or negative. When it is negative, it is a prerequisite for self-organization [2,3,4,5], and a dissipative structure can occur.

### 1.2. Dissipative Structures According to Ilya Prigogine

Irreversible processes can lead to a new type of dynamic states of matter. Prigogine called them “dissipative structures,” which represent a form of order arising from non-equilibrium conditions [6,7]. The spontaneous appearance of ordered structures appears as a result of irreversible processes maintained by exchanges of matter and energy with the environment. Dissipative structures generally evolve on macroscopic scales and can only be maintained by continuous application of a non-equilibrium constraint. Their spontaneous emergence arises from a principle of self-organization that can operate in space and/or in time. They denote a structure within a physical system that is actively maintained by a flow of energy and/or matter, rather than being an inert structure that is merely resistant to decay.

**Necessary conditions**: There are conditions for dissipative structures: the system must be open, governed by nonlinear evolution equations, and operating far from thermodynamic equilibrium. Three aspects are always linked in dissipative structures: the function as expressed by chemical or physical equations; the space–time structure, which results from instabilities; and the fluctuations, which trigger those instabilities. The interplay between these three aspects leads to highly unexpected phenomena, including “order through fluctuations” [8].

**Bifurcations**: When a characteristic parameter is increased beyond critical values, the system undergoes successive bifurcations, branching into qualitatively different states of organization [9].

**Self-organization and irreversibility**: These open systems achieve stability through energy dissipation and self-organization, leading to complex structures from disorder. The emergence of order from chaos highlights the irreversibility of time, blending deterministic and stochastic processes [10].

**Examples of dissipative structures**: Examples of dissipative structures include the Bénard–Taylor convection cell, turbulence, hurricanes, vortices [9]; Turing structures [11]; Belousov–Zhabotinsky reactions [12]; chirality; enantiomers and racemization [9,13]; biological rhythms [14]; and allosteric systems [15]. In medicine, dissipative structures are not very well known. Since the work of Ilya Prigogine, which earned him the Nobel Prize in Chemistry in 1977, they have become much more widely recognized in the fields of physics, chemistry, and even biology [16]. They have been described as being involved in cancer [17], bipolar disorder [18], medical tribology [19], myocardial hypoxia [20], and in the uterus outside pregnancy [21]. The term “dissipative structure” means that, far -from -equilibrium, in systems traversed by flows of energy and matter, spontaneous organizational processes can occur. The significance of this discovery is that irreversibility, far -from -equilibrium, can play a constructive role and become a source of order.

## 2. Methods and Results

The study reported here was conducted on human lung tissues. The following etiologies were recorded [22]. Pulmonary cancers (n = 16) and other pulmonary etiologies (n = 19): pulmonary hamartoma, pulmonary sarcoidosis, pulmonary tuberculosis, pulmonary aspergilloma, pulmonary nodular lymphoid hyperplasia, pneumoconiosis, bullous dystrophy, pulmonary paraganglioma, benign congenital cyst, chronic sero-fibrinous pleurisy, mesothelial-to-mesenchymal transition, benign mesothelial hyperplasia, adenomatous hyperplasia, nonspecific chronic fibro-inflammatory changes, organizing inflammatory and fibrous pneumonitis.

### 2.1. Histopathology and Immunohistochemistry

Tissue specimens are fixed in 10% buffered formalin and embedded in paraffin. Standard 4 μm-thick sections are stained with hematoxylin, eosin, and saffron (HES). Immuno-histochemistry is performed on Ventana Bench Mark Ultra using the primary polyclonal antibodies MYH9 (NM-IIA, FineTest (Palm Coast, FL, USA), dilution 1:100; catalog no. FNab05479-20) and MYH10 (NM-IIB, FineTest, dilution 1:100; catalog no. FNab05471), the mouse monoclonal antibody β-catenin (β-catenin, Cell marque (Rocklin, CA, USA), dilution 1:25; catalog no. 224M-14), and smooth muscle actin (SMA clone 1A4, Zytomed Systems (Berlin, Germany), dilution 1:100). See Figure 1, Figure 2 and Figure 3.

### 2.2. Contractile Function and Thermodynamic Properties

The following step is to determine the contractile properties of the lung fragments [23]. Normal lung parenchyma does not exhibit contractile properties. In contrast, pathological lung parenchyma acquires contractility through the synthesis of a myosin—specifically non-muscle myosin—such as in inflammatory states [22]. Maximum shortening velocity (Vmax) and maximum isometric tension do not differ between lung cancers and other pulmonary etiologies (Figure 4A,B). G curvature (Figure 4C) and time stroke (Figure 4D) do not differ in both groups.

The initial objective is to determine the entropy production rate (y = EPR). This is the quantity necessary to initiate the calculations. Starting with living tissue possessing a contractile function, it is possible to calculate the energy generated inside a tissue. The mathematical formalism given by A. Huxley’s equations [24] allows us to determine the power and, after integration, the energy produced by the contractile tissue. Muscles, with their molecular motor, muscle myosin (M), offer the possibility of applying this method. But there are other living tissues, i.e., non-muscles, with their molecular motor, non-muscle myosin (NM), offering the same possibility. These tissues possess all the contractile properties of muscles [22]. Huxley’s equations are applicable to them and are a powerful tool to characterize the molecular properties of contractile proteins, in particular those of crossbridges (CB) [24,25,26]. The rate of total energy release (E_Hux_’) as a function of velocity (V) was obtained by the following equations:

E_Hux_’ = (N e) (h/2 l) (f1/(f1 + g1)) {g1 + f1 (V/Φ [(−exp (−Φ/V)]} (1)

In that equation, f1 corresponds to the maximum value of the rate constant for NM-CB attachment; g1 and g2 represent the maximum values of the rate constants for NM-CB detachment; f1 and g1 correspond to the tilt of the CB from 0 to h; and g2 corresponds to a tilt > h. The NM-CB myosin tilt relative to the actin filament varies from 0 to h. This represents the distance of translocation of the actin filament after the NM-CB swing. The constant l is the distance between two successive actin sites with which a myosin head can bind. A. Huxley conditions impose that l >> h; h = 10 nm and l = 28.6 nm [24]. Moreover, w is the maximum mechanical work generated by a unitary NM-CB; e is the free energy to split one ATP and is equal to 10^−19^ J; w/e = 0.75. The standard free energy ΔG°’ATP is −60 kJ/mol; Φ is equal to (f1 + g1) h/2 = b; N is the NM-CB number per cross-sectional area.

The parameters f1, g1 and g2 are calculated using the following equations [27]:

f1 = Gg1(2)

g1 = 2wb/ehG(3)

g2 = 2Vmax/h(4)

The Hill hyperbolic relationship by A. V. Hill is constructed by means of contractions of increasing isotonic tension (T), from the zero-load clamp to the isometric tension. This relationship is fitted by a Hill hyperbola: (V + b) (T + a) = b (To + a). The asymptotes of the hyperbola are −a and −b. The G curvature of the hyperbola is Vmax/b = To/a (To: total isometric tension).

The sliding velocity of NM-myosin along actin filaments was vo = h/ts (in μm/s), where ts is the time stroke. PA2 is the probability of the time stroke during which the unitary NM-CB force and the swing of the NM-CB occur and is the quotient of ts over tc; tc is the duration of the entire NM-CB cycle. PA2 is equal to (h/e) po and tc = 1/kcat. The rate of total energy release E_Hux_’ under isometric conditions is equal to ab, the product of asymptotes. Thus, after integration, the thermodynamic force is E/T and its dimension is energy/T. The thermodynamic flow is the shortening velocity of the NM-myosin molecular motor (vo).

### 2.3. General Approach of Statistical Thermodynamics

The product of the thermodynamic force and the thermodynamic flow is the entropy production rate (y = EPR):

y = EPR = δ^2^S = (1/T) × E × vo (5)

The stability condition is given by the relationship

y’ = EEP = (1/T) × δE × δvo > 0(6)

The unstable condition is

y’ = EEP = (1/T) × δE × δvo < 0(7)

where y’ = EEP = $\frac{\partial \delta^{2}S}{\partial t}$ is the excess entropy production (EEP).

δE and δvo are the deviations of the thermodynamic force and the thermodynamic flow from the stationary state, respectively. When the deviation goes beyond a critical value, the system reaches an instability threshold. A transition to a self-organized structure is then possible. This is a prerequisite for self-organization [2,4,5,16].

The first group consisted solely of pulmonary cancers. The second group comprises various etiologies, excluding cancer. We check that the living tissues exchange energy and matter with the outside world. This is the case with living samples. We then study the distance from equilibrium by determining the relationship between the thermodynamic force and the thermodynamic flow. The system is close to equilibrium if there is a linear relationship between these two parameters. Otherwise, it is far-from -equilibrium if there is a non-linear relationship between them.

Thermodynamic force is slightly greater in non-cancerous inflammatory pulmonary tissues than in purely cancerous lung tissues, while thermodynamic flow does not show significant differences between the two groups (Figure 5). Above all, there is no linear relationship between thermodynamic force and thermodynamic flow (Figure 6). This means that the two lung groups are in the far-from-equilibrium domain. Entropy production rate (y = EPR) and excess entropy production (y’ = EEP) (Figure 6) do not show any significant differences. What is crucial is that EEP is negative in both groups, meaning that the two lung groups are in the self-organized domain.

### 2.4. Bifurcations and Symmetry Breaking

In Prigogine’s dissipative structures, bifurcations represent symmetry breaking [28]. Non-equilibrium thermodynamics highlights the relationship between loss of stability, the multiplicity of solutions and symmetry. It is within this framework that we encounter the phenomenon of “bifurcation”, the branching of new solutions to a differential equation, to describe far-from-equilibrium systems. Dissipative structures create “symmetry breaking” as a signature of bifurcation in Prigogine’s theory. The fundamental link between loss of stability and symmetry lies in the phenomenon of bifurcation. Before bifurcation, the thermodynamic branch possesses all the symmetries imposed by the constraints. At the critical bifurcation point, this solution becomes unstable.

### 2.5. Determination of a One-Dimensional Non-Linear Equation with One Parameter (G)

A polynomial regression between y’ and y is applied to obtain a non-linear differential equation. A term of this equation is related to y and is introduced in the differential equation. A one-dimensional non-linear differential equation with one parameter ((G) is obtained.

y’ is expressed as a function of y according to the following equation (Figure 6):

y’ = 674 − 3.2 y − 1.1 × 10^−4^ y^2^

Furthermore, y is a non-linear power function of G (Figure 6C):

y = f (G) = 17698 G^−3.8^

Thus, y’ = 674 − (3.2 × 17698) G^−3.8^ − 1.1 × 10^-4^ y^2^

**y**’ **= −1.1** × 10**^−4^ y^2^ − 57** × 10**^3^ G^−3.8^ + 674**

A one-dimensional non-linear equation is obtained with one parameter (G).

### 2.6. Phase Diagram and Bifurcation Diagram

We study the phase diagram y’ = f (y, Ψ) (Figure 6B) and the bifurcation diagram (y = f (Ψ) (Figure 6C). On the phase diagram, y’ = f (y, Ψ) shows a non-hyperbolic fixed point (y*) because λ = f’ (y*) is equal to 0. A bifurcation occurs at the non-hyperbolic fixed point.

### 2.7. Stability Analysis

The stability analysis is best determined on a case-by-case basis, using a graphical method (Figure 6C). The system has a fixed point y* with λ = f‘(y*) = 0. The slope of the function y’ = f (y, Ψ) at the fixed point y* determines the stability of the system. Thus, y* is a non-hyperbolic fixed point. Figure 6C shows that the system is unstable, and this arises in the context of bifurcation.

## 3. Discussion

### 3.1. Self-Organized Dissipative Structures

In this study, the inflammatory syndrome associated with all the described pulmonary etiologies acquires contractile properties through the hyperactivation of the non-muscle myosin. This enables the calculation of their energy and entropy. The thermodynamic system operates far -from -equilibrium because the thermodynamic flow ceases to be linearly related to the thermodynamic force (Figure 6A). Statistical entropy measures the system’s state of organization and describes its evolution at a given moment; it does not drive, cause, or affect the system’s evolution. That role falls to the rate of entropy production, which quantifies the irreversible chemical processes within the system [9]. In non-muscle tissue, the vast number of non-myosin molecules involved in contractile processes provides the necessary framework for applying statistical mechanics. This allows to establish a strong link between macroscopic and microscopic thermodynamic properties [29]. Statistical mechanics makes it possible to calculate the rate of entropy production that quantifies irreversible chemical processes. Applying statistical mechanics to living non-muscle tissues requires the use of A. Huxley’s equations [24], which allows for establishing a relationship between the non-muscle myosin kinetics and the whole non-muscle mechanics. This provides the cornerstone of using statistical mechanics as a tool to mathematically solve Huxley’s phenomenological formalism. Dissipative structures evolve toward an ordered state under the action of slight fluctuations [7,8]. They are far-from-equilibrium open systems, exchanging energy and matter with the external environment.

Dissipative structures can arise in both contractile muscle tissues and contractile non-muscle tissues. These tissues are distinguished by their molecular motors: muscle myosin in the case of muscles, and non-muscle myosin in non-muscle tissues [30]. For instance, in rat cardiac striated sarcomeric muscle, a self-organizing dissipative structure emerges during hypoxia sustained for more than three hours [20]. Human uterine smooth muscle operates far-from-equilibrium, behaving as a self-organized dissipative structure; it moves closer to equilibrium at the time of childbirth, only to revert to a far-from-equilibrium state postpartum [21].

### 3.2. Inflammatory Syndrome Generating a Dissipative Structure

Inflammatory tissues can arise from a wide variety of etiologies. However, these share a common designation coined “dissipative structures” by Ilya Prigogine. Examples of such structures abound in the physical and chemical sciences as well as in biology [9]. In medicine, they are also encountered in certain diseases, yet they had never previously formed the basis of a clinical syndrome. It turns out that this clinical syndrome is extremely common and can affect all human organs. Cancer affects virtually every organ. Tuberculosis is not limited to lung tissue but also affects the kidneys, bones, lymph nodes, genitourinary tract, central nervous system, and so on. Sarcoidosis is found in the lungs, lymph nodes, kidneys, skin, and eyes, but also in the heart, among other areas. Further experiments are needed to show that these diseases are self-organized dissipative structures.

### 3.3. Non-Exhaustive Diseases Involving an Increase in at Least One of the Biological Parameters of the Inflammatory Syndrome

There is a considerable number of diseases characterized by the hyperactivation of the WNT/β-catenin pathway, non-muscle myosin and α-SMA in newly formed myofibroblasts. This is associated with a wide range of inflammatory syndromes that can affect any organ. The organization into a contractile system stems from the hyperactivation of non-muscle myosin, giving rise to a tissue for which energy (E), and consequently the rate of energy production (E/T.vo), can be calculated. We provide non-exhaustive bibliographic examples involving WNT/β-catenin [31,32,33,34,35,36,37,38,39,40], non-muscle myosin [41,42,43,44] and α-SMA [45,46,47,48] in newly formed hyperactivated myofibroblasts [45,49,50,51,52,53,54,55,56,57,58]. Further studies will be required to determine whether these inflammatory syndromes are dissipative self-organizing structures in diseases other than pulmonary pathologies, as observed in lung diseases.

## 4. Conclusions

Inflammatory syndrome includes pain, swelling, redness and heat, as described in the semiological tetrad of Aulus Cornelius Celsus. The combined hyperactivation of WNT/β-catenin, non-muscle myosin and α-SMA characterizes the newly formed myofibroblasts. It is non-muscle myosin that imparts contractile properties to the various clinical conditions capable of developing an inflammatory presentation. In pulmonary origin inflammation, the excess entropy production observed across all etiologies (lung cancer, tuberculosis, pulmonary sarcoidosis, lung infection, etc.) is negative, characterizing them as self-organized dissipative structures.

## Figures and Tables

**Figure 1:**
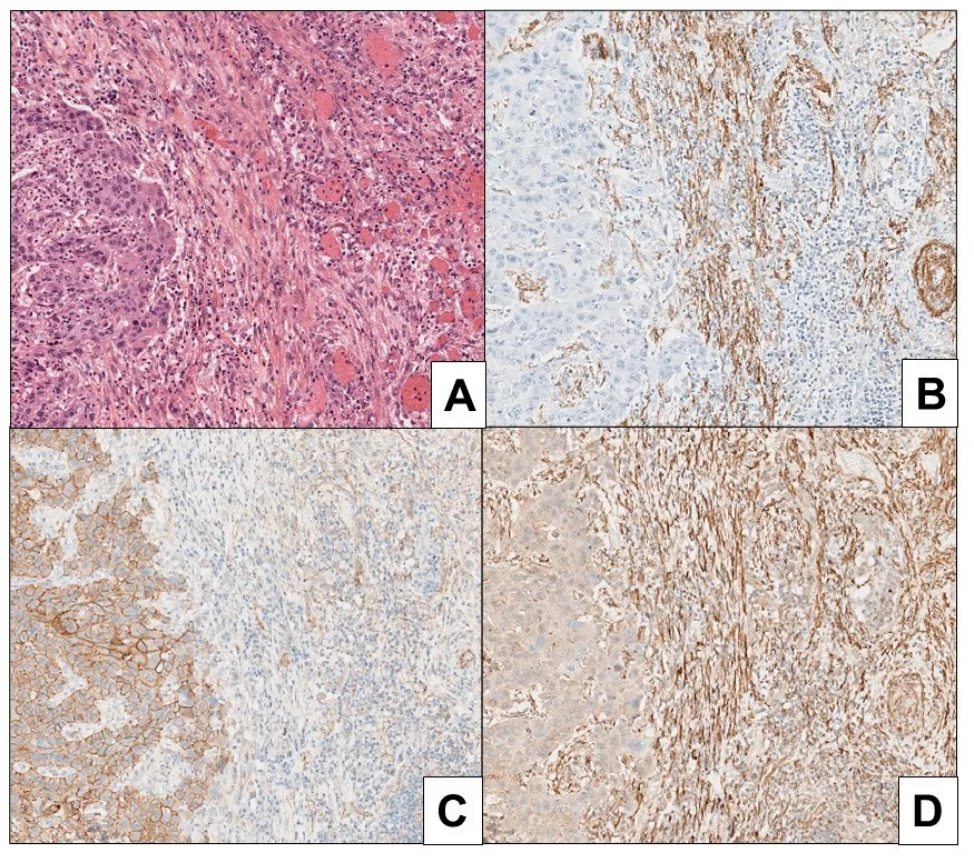
Perilesional stroma of an epidermoid carcinoma. Perilesional stroma of an epidermoid carcinoma, stained with HES (**A**), with anti-SMA (**B**), anti-β-catenin (**C**), and anti-MYH10 (**D**) immunostaining. Magnification 5× for all images.

**Figure 2:**
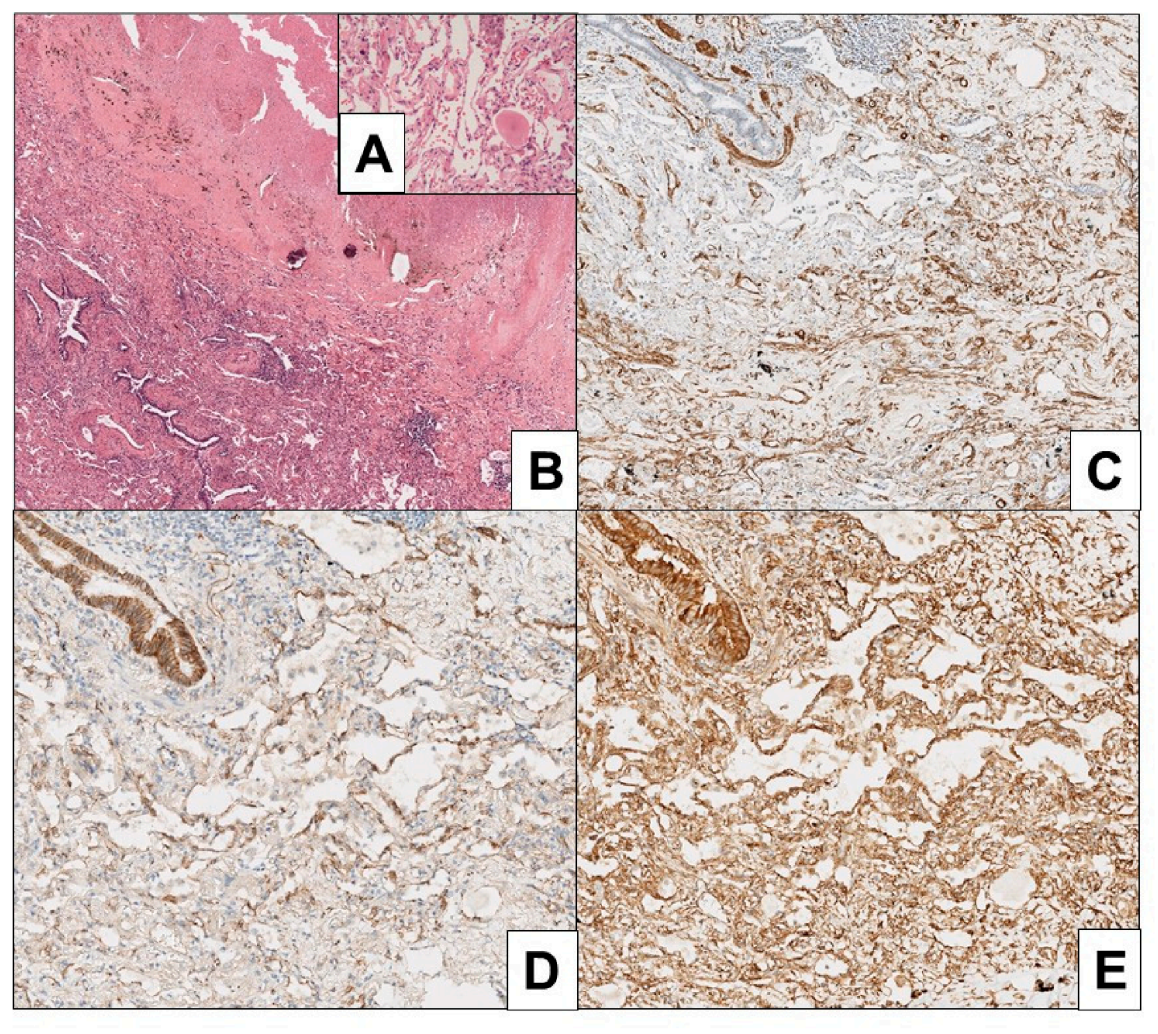
Perilesional stroma of an aspergilloma. Perilesional stroma of an aspergilloma, stained with HES (**B**) and showing a cluster of Aspergillus filaments in HES (**A**), with anti-SMA (**C**), anti-β-catenin (**D**), and anti-MYH10 (**E**) immunostaining. Magnification 2.5× for the HES image and 5× for all immunostaining.

**Figure 3:**
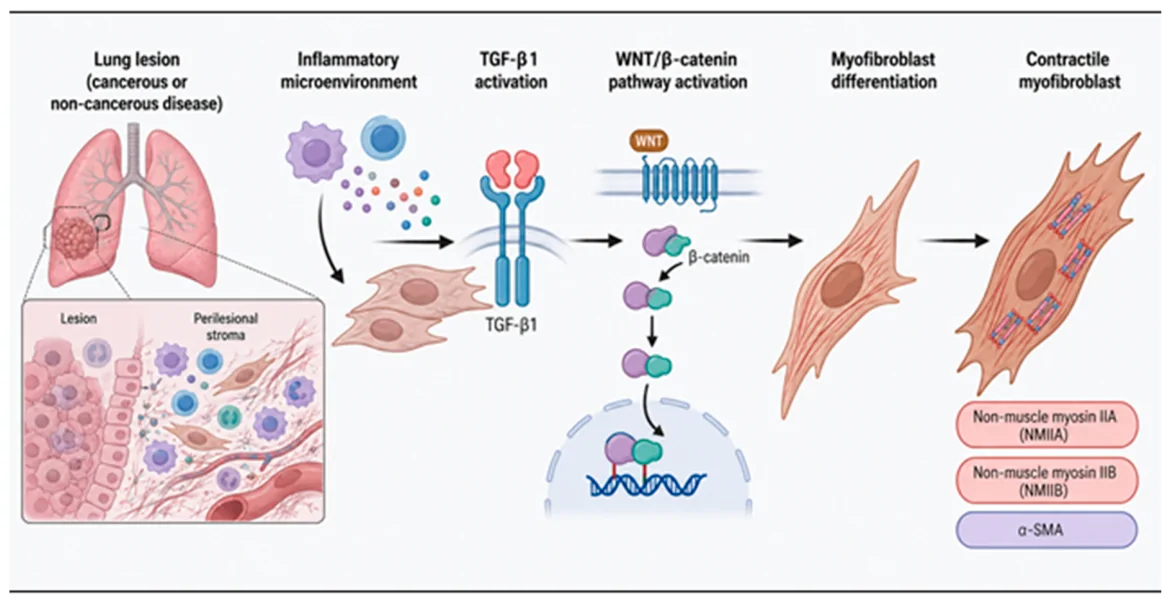
Schematic of the WNT/β-catenin, NM-myosin, and α-SMA pathways in newly formed myofibroblasts. Formation of a contractile perilesional stroma in lung lesions is under the influence of the inflammatory microenvironment; TGF-β1 and WNT/β-catenin pathways promote fibroblast-to-myofibroblast differentiation. Newly formed myofibroblasts acquire a contractile phenotype characterized by the expression of α-SMA and non-muscle myosin II isoforms (MYH9/NMIIA and MYH10/NMIIB), generating an organized actomyosin network.

**Figure 4:**
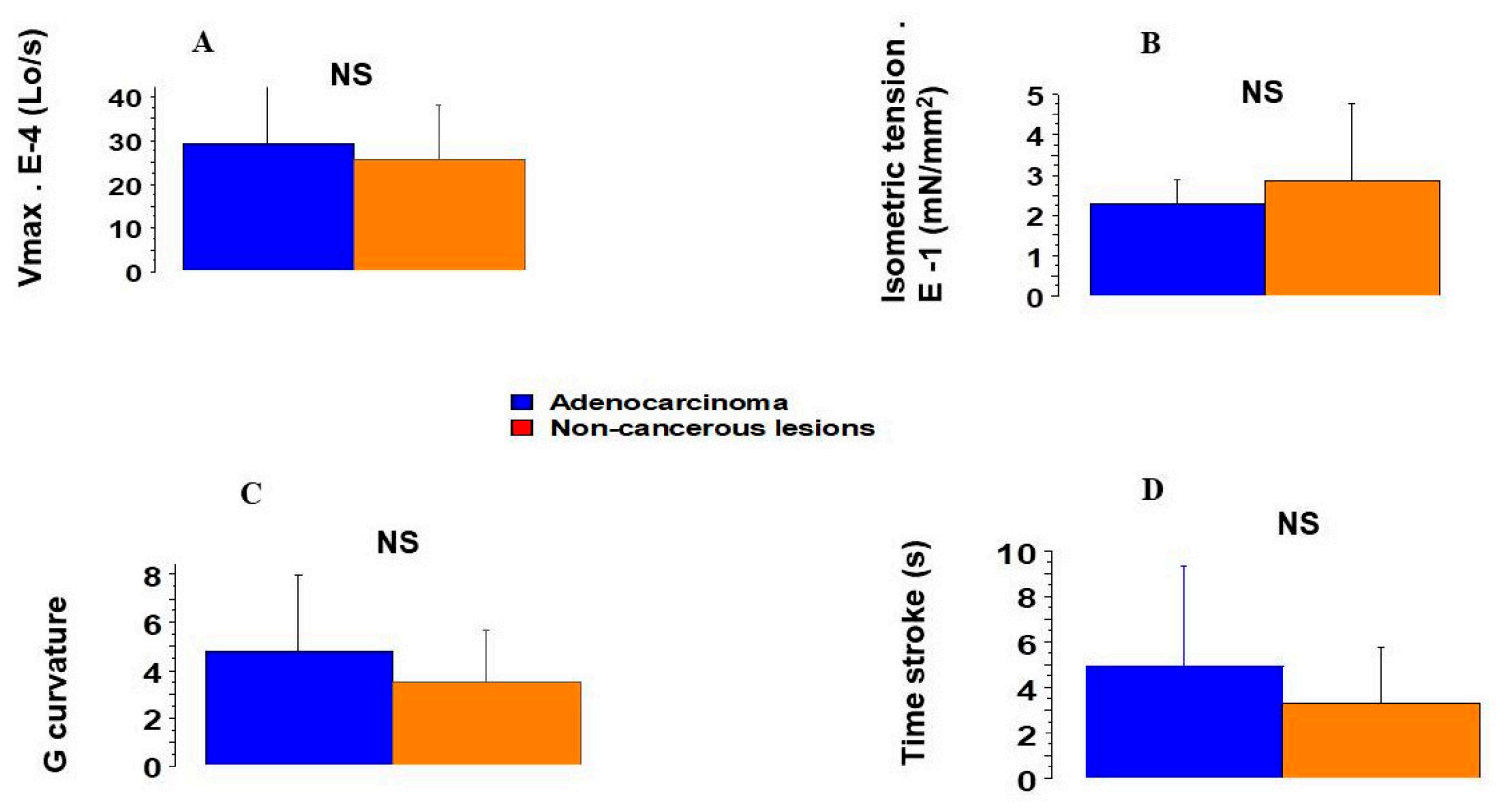
Mechanical properties of lung fragments. (**A**) Maximum unloading shortening velocity (Vmax); (**B**) total isometric tension; (**C**) G curvature of the hyperbolic velocity–tension relationship; (**D**) time stroke of the CB cycle.

**Figure 5:**
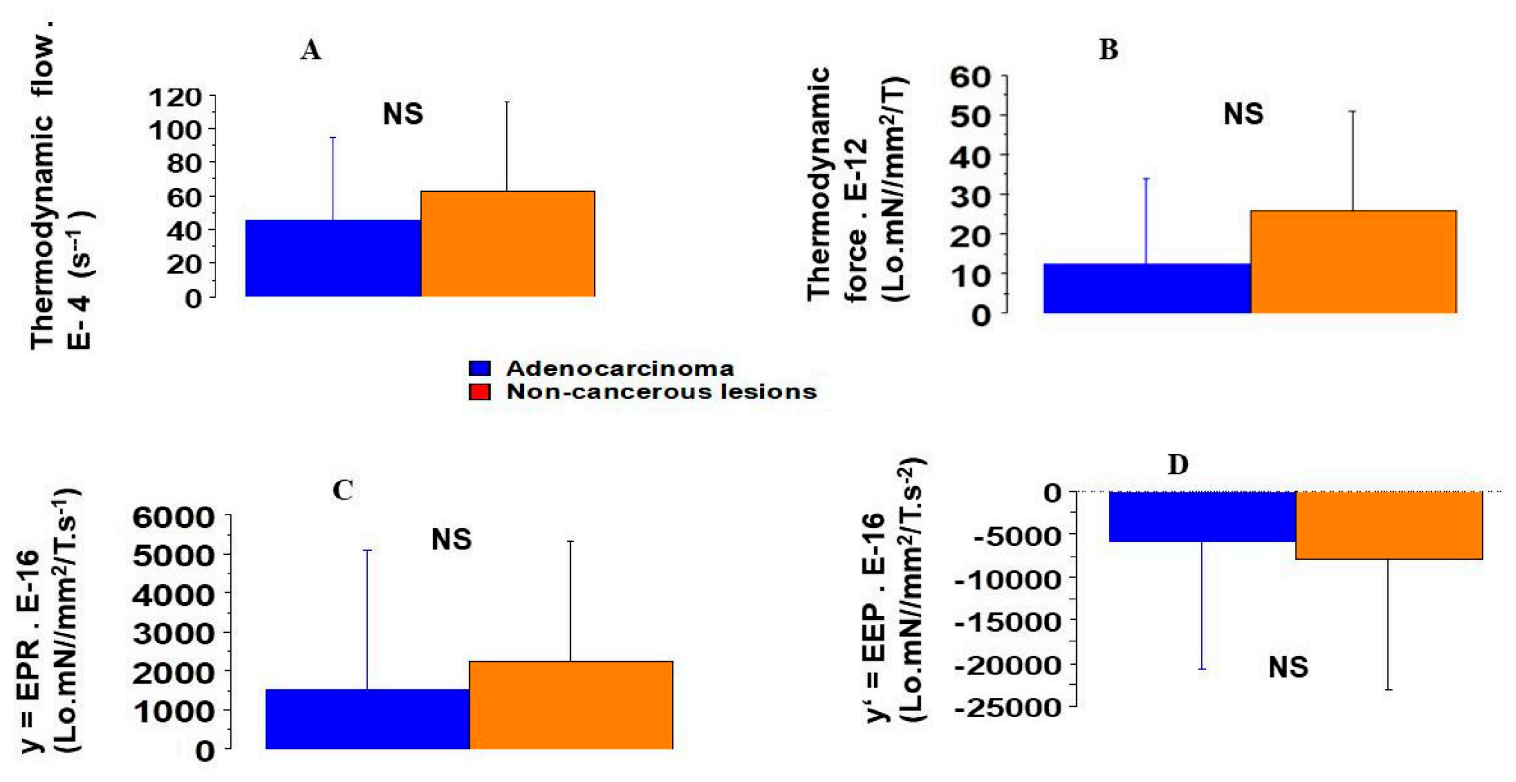
Thermodynamic parameters. (**A**) Thermodynamic flow; (**B**) thermodynamic force; (**C**) energy production rate (y = EPR); (**D**) excess entropy production (y’ = EEP).

**Figure 6:**
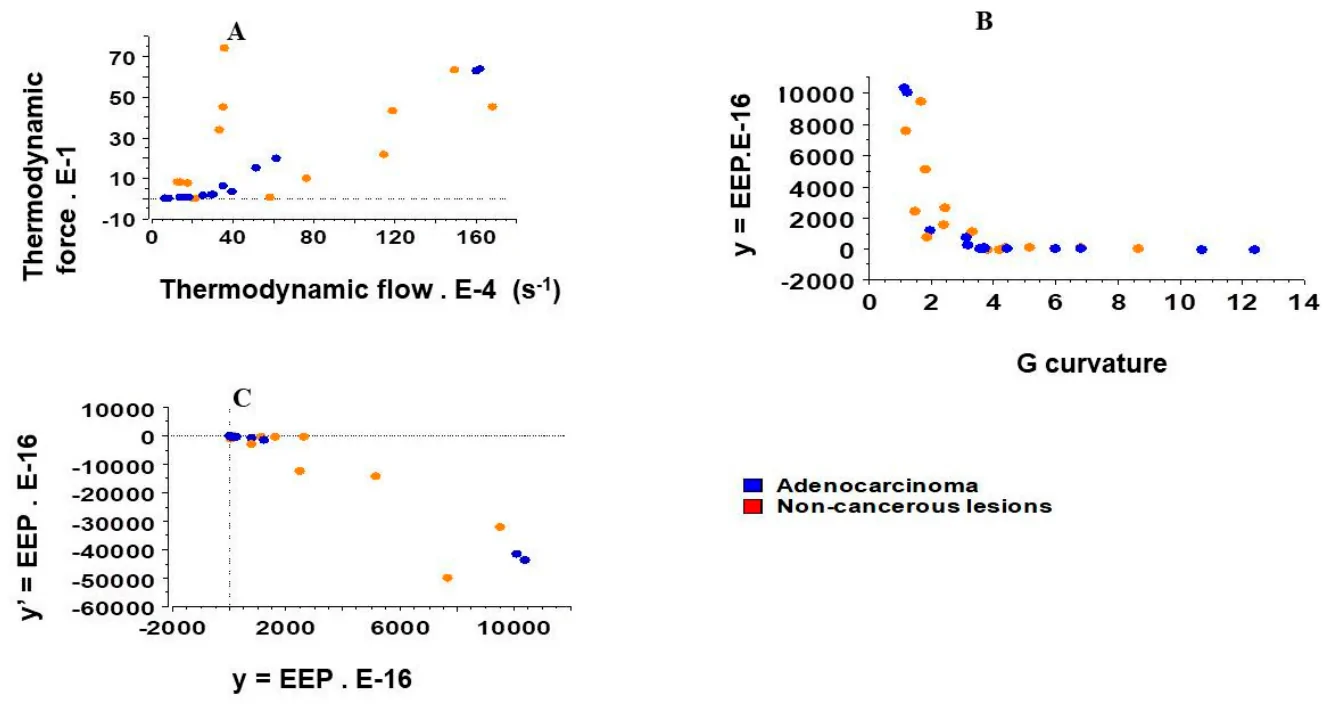
Thermodynamic parameters. (**A**) Thermodynamic force as a function of thermodynamic flow; (**B**) energy production rate (y = EPR) as a function of the G curvature, which is the bifurcation diagram; (**C**) phase diagram: excess entropy production (y’ = EEP) as a function of entropy production rate (y = EPR).

## Data Availability

The original contributions presented in this study are included in the article. Further inquiries can be directed to the corresponding author. The previously published results are primarily described in reference [22].
